# Population-based cross-sectional survey of cervical cancer screening prevalence and socio-demographic correlates in Bangladeshi women

**DOI:** 10.1007/s43999-024-00053-x

**Published:** 2024-11-06

**Authors:** Mohammad Jobair Khan, Priya Kannan, Sayma, Stanley John Winser

**Affiliations:** 1 Department of Rehabilitation Sciences, The Hong Kong Polytechnic University, Hung Hom, Hong Kong (SAR), China; 2 Rehabilitation Sciences, The Hong Kong Polytechnic University, Kowloon, Hong Kong (SAR) China; 3 Department of English, Uttara University, Dhaka, Bangladesh

**Keywords:** Bayesian regression model, Cervical cancer, Screening, Prevalence, Sociodemographic correlates, WHO STEPS survey

## Abstract

**Background:**

Cervical cancer, albeit preventable, is the second-most deadly gynecological cancer in developing nations. Little is known about cervical cancer among Bangladeshi women. This study aims to estimate the prevalence of cervical cancer screening and demographic correlates to identify potential variabilities in screening rates among different demographic groups and regions.

**Methods:**

This study used secondary data from the WHO STEPS 2018 Survey. We used Bayesian regression to perform the bivariate analyses between the outcome and each explanatory factor, as it generates more acceptable results and improves parameter estimates. The top-ranked socio-demographic factors were identified using a two-step cluster analysis. This method determines the relevance of predictor variables and automatically establishes the number of clusters.

**Results:**

The prevalence of Bangladeshi women who had ever been screened for cervical cancer was 6.2%. In the adjusted model, women with the following socio-demographic factors had a higher likelihood of developing cervical cancer: being 18–29 years old (AOR = 3.3, 95% CI: 0.24, 15.27) or 45–59 years old (AOR = 2.8, 95% CI: 1.22, 6.0), currently married (AOR = 2.3, 95% CI: 1.36, 3.70), and employed (AOR = 2.4, 95% CI: 1.40, 4.06). Women in the Barisal division were found to have higher odds of being screened for cervical cancer (AOR = 21, 95% CI: 0.66, 121.97). Cluster analysis found residence status predisposes women to cervical cancer screening.

**Conclusion:**

There is a significant potential for substantial reductions in the burden of cervical cancer in Bangladesh by strengthening the application of cervical cancer screening. Future studies should examine how socioeconomic status, culture, and healthcare access affect cervical cancer screening trends for different divisions in Bangladesh. An independent national cancer registry is urgently needed to evaluate screening trends and outcomes.

## Introduction

Globally, cervical cancer is the fourth most common cause of death in women [1]. Every year, over half a million women are identified as having cervical cancer, resulting in 300,000 deaths [2]. Nearly 90% of deaths occur in low- and middle-income countries due to inadequate screening or human papillomavirus immunization programs [2, 3]. Despite the decrease in the number of deaths from cervical cancer, 13.3 out of every 100,000 women die in low- and middle-income countries [4, 5]. The age-standardized incidence, prevalence, and death rates of cervical cancer due to human papillomavirus were 10.6, 7.6, and 6.7 per 100,000 women, respectively, in 2023 [6]. In the South Asian Association for Regional Cooperation region, the highest cervical cancer incidence was reported in India at 18.7, followed by Nepal at 14.2, Bangladesh at 10.2, and Pakistan at 4.7 per 100,000. The age-standardized cervical cancer incidence rate was also higher in India at 18, Nepal at 16.4, Bangladesh at 10.2, and Pakistan at 6.1 per 100,000 [7–10]. The mortality rate bordering Bangladesh, in Myanmar, recorded the highest at 14.5 per 100,000 and India at 12.4, while regional country Pakistan at 5.2, and Bangladesh lowest at 5.1 [11–14]. The published study did not consider the other risk factors for cervical cancer, which does not reflect the overall scenario in Bangladesh [6].

Evidence shows that early screening and treatment can limit one-third of cervical cancer cases [15]. The reasons for cervical cancer are complicated, both due to aging and the increase in the female population, which have changed the prevalence and distribution of the major correlates for cervical cancer [16–18]. In 2018, cervical cancer was the second-leading cause of cancer in Bangladesh, with an incidence rate of 10.6% and a mortality rate of 5.2% [19]. Consequently, it is crucial to determine the prevalence of cervical cancer screening using an updated and comprehensive dataset.

Sociodemographic factors such as age, residence, division, education level, and marital status are critical in shaping health outcomes. By examining these variables, identify vulnerable populations that are out of the existing scope of the cervical cancer screening facility. Lower education levels often correlate with reduced awareness and access to preventive measures like Pap smears and HPV vaccinations, leading to higher incidence rates [20]. Marital status influences exposure to HPV, the primary cause of cervical cancer, making these factors crucial for targeted interventions [21]. It is essential to note that the incidence of cervical cancer significantly differs between rural and urban areas. Study reported cervical cancer rates are notably higher in rural areas than in urban areas [22]. Almost a decade ago, a survey undergone in Bangladesh on cervical cancer excluded women under 30 and did not analyze administrative regions for cervical cancer correlates [23]. Understanding sociodemographic factors is not just important, but it is crucial for designing better-targeted interventions. This understanding enhances the effectiveness of public health strategies for cervical cancer. The study can identify high-risk groups by analyzing sociodemographic factors. This analysis also reveals barriers to healthcare access, informing the design of interventions such as establishing more screening units and community health worker programs. Sociodemographic data not only helps allocate resources efficiently and engage community leaders to support public health initiatives but also plays a key role in promoting health equity, a crucial aspect of social justice in healthcare.

Recently published, a single-center-based lower number of participants in recruitment does not reflect the representative results of the country as a whole [24]. Additionally, convenience sampling procedures have been adopted, which causes a higher possibility of researcher bias in recruiting respondents [24]. As a consequence, it is obligatory to conduct a widely generalizable study to identify the other sociodemographic correlates with cervical cancer. Therefore, the purpose of this study is to investigate the screening prevalence of cervical cancer among Bangladeshi women and the sociodemographic factors that correlate with it using nationally representative data. The outcome of this nationally representative study certainly aids in identifying the most vulnerable groups with sociodemographic correlates, which can be appropriately utilized in national policymaking.

## Methods

### Participants and public involvement

This study was a secondary analysis of a prospective study comprising 4381 Bangladeshi women using the WHO STEPS 2018 Survey [25].

### WHO STEPS 2018 Survey

The WHO Non-communicable Disease Risk Factor Surveillance (STEPS) system is simple and standardized for collecting, analyzing, and distributing data on key risk factors in nations. In 2018, STEPS was used in Bangladesh for a national cross-sectional population-based survey. The survey sampled households and adults using a multi-stage cluster sampling method and considered both biological and behavioral risk factors [25].

### Study design, sampling, and period

2018, the WHO STEPS study utilized a multistage, nationwide cross-sectional study. Data were obtained from February to May 2018. Data were collected by the Bangladesh Bureau of Statistics through 496 primary sampling units (PSUs) located across Bangladesh. A probability-based sampling procedure was applied following regional stratification.

### Participant criteria for inclusion

Participants were included in the study if they (1) were in the 18–69 age group, (2) lived in their current home for at least six months, and (3) slept at home at night right before the survey. The study excluded women who resided in military bases or group quarters, jails, hospitals, nursing homes, or other institutions, including those who were deemed to be frail or mentally or physically unfit.

### Sample size

Sample size calculation was performed to ensure the generalizability and reliability of the study findings for all targeted participants. The calculated sample size was adequate to give valid estimates for all variables. The sample size was determined by noncommunicable disease risk factors’ prevalence, relative accuracy rate (20%), and survey practicality. The obesity prevalence determined that each group needed a total of 472 participants. The design effect was considered 2, with a 10% nonresponse rate for participants and a 10% noncoverage rate for households. Therefore, the total targeted sample size was 9900 participants from 495 PSUs.

### Administrative region of Bangladesh

The administrative system of Bangladesh is divided into eight divisions at the first-order administrative level. These eight divisions are Barisal, Chattogram, Dhaka, Khulna, Rajshahi, Rangpur, Mymensingh, and Sylhet. Each division is named after the most significant city located within its borders, which is also the administrative capital of that division. At the second-order administrative level, the divisions are further divided into 64 districts. These districts are further subdivided into around 500 sub-districts and finally into unions [26].

### Sampling frame and strategy

The sampling frame was constructed utilizing the comprehensive inventory of PSUs compiled by the Bangladesh Bureau of Statistics (BBS). It included pertinent details such as PSU location, residential classification, and estimated number of households. All 2,93,533 PSUs were mapped for the study. There were 6,51,93 urban PSUs and 2,28,340 rural PSUs. Households were selected from BBS’s updated household lists. Twenty households from each PSU were randomly selected and assigned “male” or “female” in a ratio that created a comparable percentage of male and female households. One person was randomly chosen for sampling among all the eligible individuals in a household. It was not permitted to replace or alter the preselected households during the implementation phase to prevent bias.

The PSUs were distributed evenly across the divisions (62 for each) and among the urban and rural strata (248 PSUs each). PSUs were classified based on population size, as measured by the number of households, for both the urban and rural strata. Using probability proportional to size sampling, 31 PSUs were randomly chosen from each stratum and then placed into each division.

### Data collection, quality control, and management

Data were collected using a pretested and standardized WHO STEPS questionnaire (version 3.2). The questionnaire includes core questions as well as additional extended and country-specific items. The Bengali questionnaire underwent verification through translation and subsequent reverse translation. The initial phase of data collection involved conducting in-person interviews. The data acquisition process on Android devices was performed on-site and subsequently transferred to the cloud through ODK software. The quality control process involves daily examination of data and monitoring field operations.

The ODK software was utilized for data entry on handheld electronic devices. The database server electronically received the information. The field crew uploaded data on a daily basis to the server. The central office downloaded the data to Microsoft Excel for consistency and validity checks. Data integration was facilitated by utilizing the QR code and personal identification number assigned to each participant.

The data cleaning process adhered to the WHO STEPS guidelines. The data cleaning process involved four steps: (1) Generating a unique participant ID (PID); (2) determining location variables such as cluster ID and cluster name; (3) reviewing open-ended questions; and (4) resolving errors by double-checking location variables. The same PID was required of a participant across the study period. Incorrect PIDs hindered the process of creating cluster IDs. To resolve this error, double-check location variables, follow up with the field team supervisor(s), and communicate with the relevant data collector for further clarification. If required, request further clarification from participants. Further details have been published elsewhere [27].

### Measures

#### Outcome variable

The study was intended to determine the prevalence of cervical cancer screening and its associated factors. As a result, this variable in the dataset was chosen as “even had cervical cancer screening test.” There are three possible answers to choose from when responding to this variable: “Yes,” “No,” or “Do not Know.” The responses “no” were classified as “0” in the dataset, while the responses “yes” were coded as “1”.

#### Explanatory variables

Sociodemographic factors, including age, education, marital status, occupation, place of residence, and region of residence, were determined and adopted as explanatory variables based on a review of the literature and their inclusion in this study.

#### Analysis

The analysis did not involve excluding any missing data. Thus, 4381 of 8,185 (82.7%) target women completed STEP-1 and were therefore included in the analysis. Descriptive statistics were employed to provide an overview of the sample’s factors.

Bayesian precedes frequentist statistics. Using previous information, the Bayesian methodology estimates the likelihood of a hypothesis. Unlike frequentist analysis, Bayesian analysis treats parameters and hypotheses as probability distributions and data as fixed. The concept is more natural because the data we collect is usually the only data set; therefore, statistical analysis thinking it is one of many may not make sense [28]. In addition, Bayesian regression generates more acceptable results and improves parameter estimates compared to other statistical methods [29]. Thus, this study applied Bayesian linear regression analysis to investigate the potential correlations between cervical cancer and various socio-demographic factors, such as age, education level, place of residence, occupation, and marital status. The degree of association between the risk factors was determined using the odds ratio (OR) and adjusted OR (AOR). The AOR was determined using the two variables of previously married and age 18. Outcome measures and group differences were calculated at 95% credible intervals (CIs) [30].

The socio-demographic factors such as age, residence, education, marital status, and occupational status were analyzed using a two-step cluster analysis (TSCA). TSCA differs from traditional clustering techniques like K-means and hierarchical clustering. It can define clusters based on categorical and continuous variables extracted from large datasets, specify the relevance of predictor variables, and automatically determine the number of clusters. The number of TSCA clusters was automatically computed through this process. Schwarz’s Bayesian information criterion (BIC) and the average silhouette coefficient were used to assess the model fit. The BIC was the selection criterion among a finite set of models, favoring the model with the lowest BIC value [31]. The silhouette coefficient, which ranges from 0 to 1 (with 1 being the best), was used to evaluate the internal validity of the model. A good model fit was defined by the lowest BIC value, highest BIC change, and an average silhouette coefficient equal to or higher than 0.50 [32].

Statistical significance was considered if the 95% CI value did not contain 0. The Bayesian regression used STATA version 17 (StataCorp LLC, College Station, TX, USA). The two-step cluster statistical analysis used SPSS (IBM Corp., IBM SPSS Statistics for Windows, Version 24.0, Armonk, NY).

## Results

### Regional variations of prevalence of cervical cancer

**Fig. 1:**
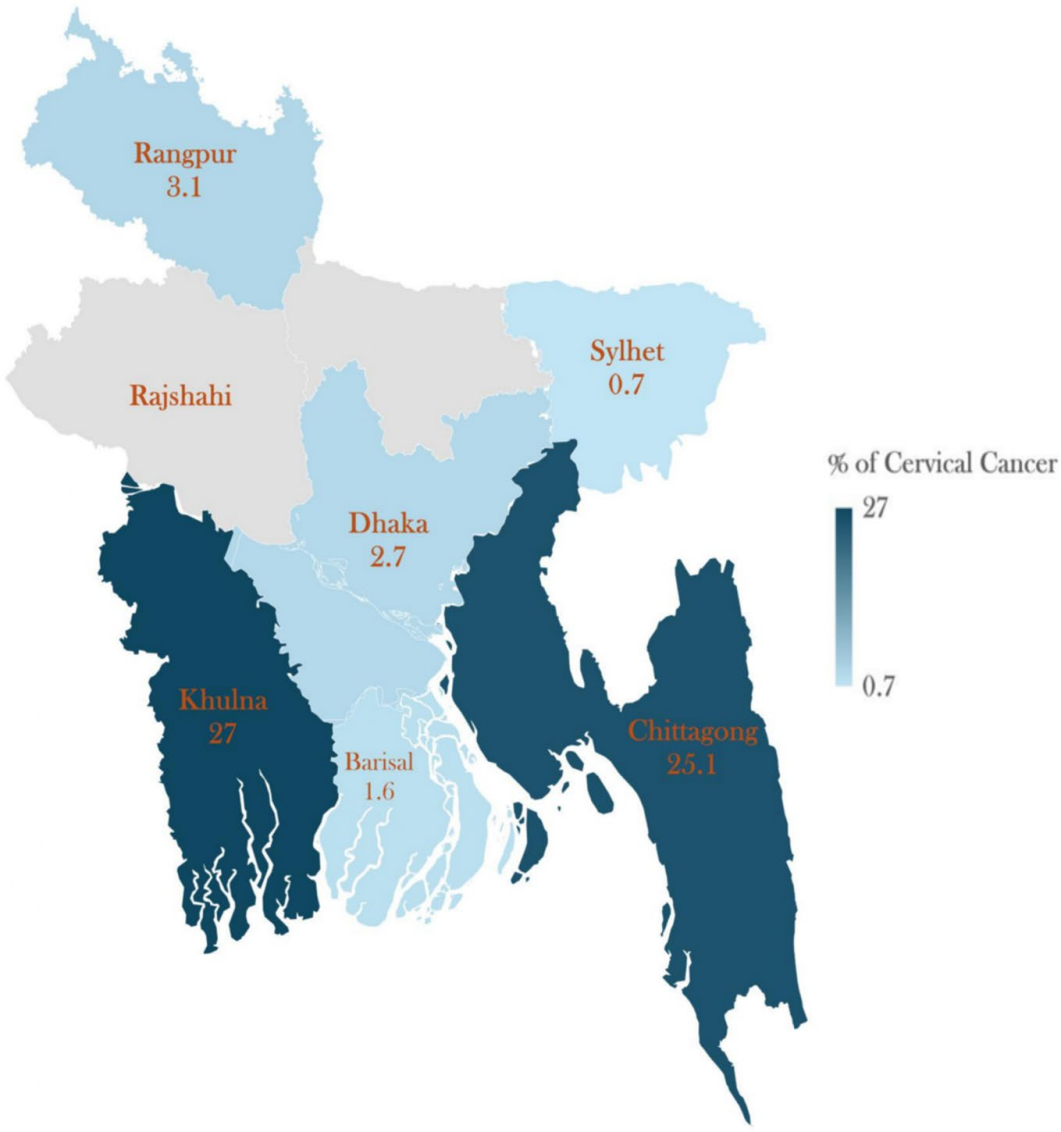
Regional differences in cervical cancer screening rates across Bangladesh

Figure 1 illustrates the regional disparities in cervical cancer screening across different divisions in Bangladesh. Khulna Division reported the highest rate of cervical cancer screening at 27%, followed by Chittagong Division at 25%, Mymensingh at 8.15%, Rangpur at 3.1%, Dhaka and Khulna at 2.7%, Barisal at 1.6%, and Sylhet at 0.7%. Table 1 provides detailed information regarding the sample description. In general, the women were of relatively young age (30–44). A considerable number of them have an education that is equivalent to or lower than that of secondary school (64.6%), are recently married (65.4%), and reside in rural areas (44%). Approximately 68.4% of the women were employed.

**Table 1:** Socio-demographic factors of the study population, 2018 (*n* = 4381)

Characteristics		Cervical cancer	
		No, %	Yes, %
**Age**	18–29	3	3.6
	30–44	20.2	33.3
	45–59	10	26.2
	60–69	1.6	1.9
**Residence**	Urban	18.8	37
	Rural	16	44.2
**Division**	Barisal	0.7	1.6
	Chittagong	7.2	25.1
	Dhaka	4	2.7
	Khulna	1.8	27
	Mymensingh	2.4	8.1
	Rajshahi		
	Rangpur	15	3.1
	Sylhet	0.3	0.7
**Education**	High School	34.8	64.6
	College/university	0	0.4
**Marital status**	Currently married	28.1	65.4
	Previously married	3.4	3
**Occupation**	Employed	30.4	68.4
	Unemployed	1	0

### Socio-demographic correlates

There was a significant association between all the study factors and the outcome factors, as shown in Table 2. The results of the adjusted model indicated that women in the age group of 18 to 29 have a 3.3 (95% CI: 0.24, 15.27) times higher likelihood of developing cervical cancer as compared with women aged 30–69. Currently married women are 2.3 times (95% CI: 1.36–3.70) more likely to report cervical cancer than previously married women (AOR = 1.9, 95% CI: 0.26, 7.19). Besides, the adjusted odds of cervical cancer are 2.4 (CI: 1.40, 4.06) times greater for employed women compared to unemployed women. In both the adjusted and the unadjusted models, the odds for women who reside in rural (AOR = 2.2, 95% CI: 1.07, 4.42) and urban (AOR = 2.2, 95% CI: 1.11, 4.13) areas with education in high school (AOR = 2.7, 95% CI: 1.51, 5.02), and college or university (AOR = 2.2, 95% CI: 1.07, 4.42) remained unchanged.

**Table 2:** Association between cervical cancer and socio-demographic factors for women in Bangladesh, 2018 (*n* = 4381)

Characteristics		OR (95% CI)	AOR (95% CI)
**Age**	18–29	3.2 0.24, 14.89	3.3 0.24, 15.27
	30–44	2 1.06, 3.75	2 1.07, 3.66
	45–59	2.7 1.16, 5.66	2.8 1.22, 6.0
	60–69	2.2 1.36, 3.45	2.3 1.34, 3.78
**Residence**	Rural	2.2 1.07, 4.37	2.2 1.07, 4.42
	Urban	2.2 1.15, 3.96	2.2 1.11, 4.13
**Education**	High School	2.7 1.50, 4.76	2.7 1.51, 5.02
	College/ University	2.8 1.51, 4.87	2.8 1.53, 5.08
**Marital status**	Currently married	2.2 1.34, 3.71	2.3 1.36, 3.70
	Previously married	1.9 0.28, 7.45	1.9 0.26, 7.19
**Occupation**	Employed	2.2 1.35, 3.53	2.4 1.40, 4.06
	Unemployed		
**Division**	Barisal	13.5 0.30, 69.30	21 0.66, 121.97
	Chittagong	1.6 0.31, 5.47	1.5 0.32, 4.85
	Dhaka	1.9 0.10, 8.93	1.8 0.10, 8.32
	Khulna	6.5 2.51, 15.70	7.1 2.79, 17.71
	Mymensingh	4.9 2.26, 9.97	5.3 2.37, 11.06
	Rajshahi		
	Rangpur	0.3 0.05, 0.82	0.26 0.04, 0.76
	Sylhet	5 0.57, 20.26	2.1 1.54, 3.10

*OR* Odds Ratio, *CI* Confidence Interval, *AOR* Adjusted Odds Ratio, *AOR* was done using Previously married and Age 18 variables

95% CI value that does not contain 0 indicates significance

### Demographic variations of cervical cancer screening in Bangladesh

The variations in the degree of correlation are more apparent when considering different demographic areas known as divisions in Bangladesh, as illustrated in Table 2. Overall, the cervical cancer screening odds were reported to be significantly lower in women who lived in Rangpur (AOR = 0.26, 95% CI: 0.04, 0.76), followed by Chittagong (AOR = 1.5, 95% CI: 0.32, 4.85), Dhaka (AOR = 1.8, 95% CI: 0.10, 8.32), and Sylhet (AOR = 2.1, 95% CI: 1.54, 3.10). Rather modest odds of cervical cancer were noted in the divisions of Mymensingh (AOR = 5.3, 95% CI: 2.37, 11.06) and Khulna (AOR = 7.1, 95% CI: 2.79, 17.71). Remarkably, higher cervical cancer screening odds for women were in the Barisal division (AOR = 21, 95% CI: 0.66, 121.97). However, in the unadjusted model, the odds of cervical cancer screening were twice as high in the Sylhet division, but this disparity disappeared in the adjusted model. On the other hand, higher odds of cervical cancer screening were reported for the Barisal division in both the adjusted and unadjusted models.

### Top-ranked factors

**Fig. 2:**
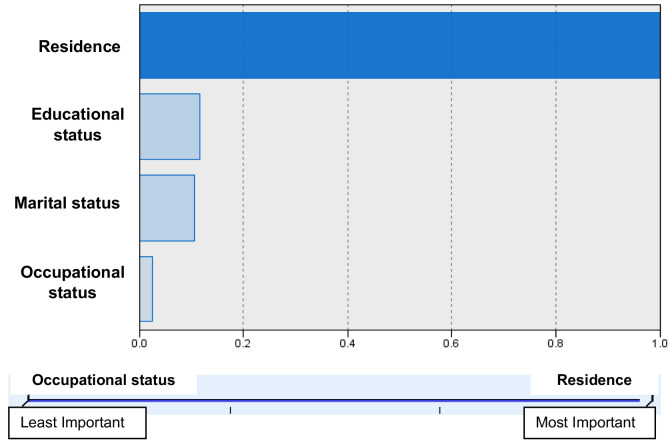
Cluster analysis of the socio-demographic factors for Bangladeshi women, 2018 (*n* = 4381)

Figure 2 depicts the rank of the socio-demographic factors of the population. Two-step cluster analysis reported that the resident was the significant predictor. The silhouette measurement was good (not shown).

## Discussion

This study is a secondary analysis of the WHO STEPS 2018 Survey. Study evaluated the prevalence and sociodemographic factors associated with cervical cancer among Bangladeshi women utilizing nationally representative data. Findings indicated that 6.2% of women of reproductive age in Bangladesh had ever been screened for cervical cancer. This screening rate is lower than that in other Asian and African countries, such as Nepal (18.3%) and Tanzania (21%), and higher than that in Sri Lanka (1.3%), India (2.6%), Ghana (3%), Cameroon (4%), Uganda (4.8%), and Kenya (6%) [13, 33–37]. Our results revealed that cervical cancer was associated with age, place of residence, divisions of Bangladesh, level of education, and marital and occupational status. Exceptionally lower adjusted odds were found among women in the Barisal division (OR: 21).

Cervical cancer was more prevalent among women aged 30–44, although the significant association was higher for those aged 18–29. Bangladesh followed the WHO guidelines for conducting cervical cancer screenings for people over 30 years of age [13]. Our study found that women in the age group of 18–29 have a higher probability of acquiring cervical cancer. It has been reported that this age group is largely devoid of cervical cancer screening procedures [38]. Policymakers in Bangladesh are encouraged to include cervical screening for women aged 19–28. Early screening for cervical cancer is reported to prevent major disease and optimize prognosis [39]. This finding is contradictory to findings in other low-resource countries, where the odds of getting screened for cervical cancer were significantly higher [17, 40]. Early screening for cervical cancer is reported to prevent major disease and optimize prognosis [39]. This finding is contradictory to findings in other low-resource countries, where the odds of getting screened for cervical cancer were significantly higher [17, 40].

The odds of undergoing cervical cancer screening considerably increased as the level of education rose. However, this finding is inconsistent with studies from low- and middle-income countries (LMIC), where the relationship between education and screening rates appears to differ. For instance, in Colombia, young women who lack any formal education had a mortality rate that was 6.4 times higher than that of their highly educated counterparts [41]. Studies conducted in low-resource countries such as Cameroon and Ethiopia found that a lower level of formal education was associated with lower utilization of cervical cancer screening services [17, 42]. It is possible that women did not learn or gain an adequate level of knowledge regarding cervical cancer, which may be the reason why higher education had almost no effect [43]. In another aspect, it was possible that women who were diagnosed with cervical cancer felt dread of social judgment and rejection, self-blame, and embarrassment with others, which in turn inhibited the dissemination of information regarding cervical cancer [44]. A recently published study undertaken in Bangladesh has found that marital status, literacy, and residency status are significantly associated with a lack of knowledge of cervical cancer [24]. To address these challenges, a comprehensive awareness initiative on a national scale was deemed necessary. This awareness program can begin by targeting different high schools and communities through advertisements on national television and in newspapers.

In this study, women who were currently married were more likely to have cervical cancer screening compared to those who were previously married. Our findings were in alignment with the study, where it was reported that previously married women have lower odds of cervical cancer screening. This finding was consistent with a prior study, which indicated that women who were previously married exhibit a reduced likelihood of undergoing cervical cancer screening [17]. Since previously married women remain away from sexual intercourse, the chances of cervical cancer are lower than those of currently married women. Additionally, women who have been married previously may be of advanced age or experience decreased sexual desire as a result of menopause. The age of women could potentially serve as a factor that contributes to the absence of sexual activity. Additional research is necessary to ascertain the underlying cause of the decreased incidence of cervical cancer among women who have been previously married.

The risk of cervical cancer among married women can be decreased by increasing awareness. Healthcare practitioners have the potential to exert a considerable influence in this case. For example, during prenatal and postnatal care, it was normal for pregnant women to visit doctors and midwives more or less often [45, 46]. Therefore, healthcare practitioners ought to take responsibility for addressing regular participation in cervical cancer screening and explaining to pregnant women how to avoid developing cervical cancer. Expanding the scope of the prevention strategy to include the education of healthcare practitioners on cervical cancer could prove advantageous in enabling them to disseminate information to the pregnant women who visit them. They can do it as part of their social responsibility, or the government might subsidize them.

In Malaysia, a middle-income country, a study found that urban residents had higher odds of developing cervical cancer compared to their rural counterparts [47]. This finding was also replicated in our study, and found that residents were the most significant predictors, including that living in both urban and rural areas contributes equally and significantly to the development of cervical cancer. However, a contrary finding has been reported in middle-income country like Cameroon, stating that cervical cancer incidence increases with an increase in income and urbanization [17]. Study argued that women living in urban areas have better access to health services than those in rural areas. Rural areas were riskier due to the lack of public hospitals that offered cervical cancer screening, which were mainly located in urban areas [17]. This issue is prevalent in low- and middle-income countries such as Bangladesh, where only a few public and private hospitals provide oncology treatment, mainly in the capital city. Earlier, the government adopted cervical cancer screening by visual inspection of the cervix with acetic acid, with only 570 centers available, which is insufficient to provide adequate services for a densely populated country like Bangladesh [13]. Therefore, it is essential to establish more cervical screening centers in the initial phase and, at the same time, develop specialized departments in public and private hospitals to admit cervical cancer patients.

This study found that demographic disparities significantly contribute to the development of cervical cancer in women. Women in the Barisal division had higher odds of cervical cancer screening compared to other regions in Bangladesh. Although Barisal was a moderately ranked division in terms of different health indicators [48], the reasons for the higher cervical cancer screening rates in Barisal are poorly known and extensively unexplored. However, a lack of knowledge and awareness among women on cervical cancer might be the major reason for the higher odds. Several studies have reported that these gaps are even more common among healthcare providers [23]. A previous study aimed at exploring the lack of understanding of cervical cancer and screening was the leading barrier to screening uptake in women at midlife in various districts, including the Barisal division in Bangladesh [23]. A recent study reported that the knowledge gap on cancer among healthcare providers still remains [49]. Although the average literacy rate among women is 59.9% [50], and the reproductive age of 15–24 is higher at 91.6% in Barisal [51], women lacking adequate knowledge and awareness of cervical cancer are likely to have higher odds of screening. Reason behind the lower and modest correlation of cervical cancer in Chittagong, Rangpur, Sylhet, and Mymensingh, including the highest correlation in Barisal, were barriers to medical screening, including lack of facilities, fear of misdiagnosis, low-risk perception, and no symptoms during cervical screening [52]. Furthermore, most of the country’s physicians practiced in the capital, which meant that women living outside the capital had limited and inequitable access to healthcare resources and facilities [17, 53]. It was possible that women who lived in the capital city of Dhaka had a low rate of cervical cancer screening for different reasons. Even though the population was well-educated and had access to sufficient information, their families’ attitudes towards screening, a lack of awareness of women’s health, high medical expenses, and/or a shortage of time could be contributing factors.

### Strengths and limitations

This study has several strengths: (1) larger sample sizes; and (2) participants were recruited from all eight divisions of Bangladesh, which is nationally representative, suggesting that our results are generalizable for the entire country.

Our study has some flaws: (1) we did not establish cause-and-effect relationships between cervical cancer and risk factors because it only provides information about the prevalence or distribution of exposure and its outcome at a single time; (2) this study only considered the prevalence of obesity when estimating the sample size, which may not represent the prevalence of noncommunicable diseases for all risk factors. To include diversity in the study population, future studies require adjusting for the prevalence of noncommunicable disease risk factors when estimating the sample size; (3) selection bias, as adults who choose to participate may be different from those who choose not to participate; (4) sampling bias may remain as the study did not include women living in specific military bases, group quarters, jails, hospitals, nursing homes, or other institutions; (5) economic status, access to healthcare facilities, and cultural beliefs may be confounding factors in our study. Future research should explore additional factors influencing cervical cancer screening uptake, such as cultural beliefs, socio-economic status, and healthcare accessibility; and (6) the accuracy of measurements can be a concern in our study, as exposure and outcome data are collected at the same time and self-reported data may be subject to recall bias.

### Implications

To the best of our knowledge, Bangladesh lacks an autonomous national cancer registry agency or a dedicated institution for cervical cancer screening. This absence is critical for evaluating future trends and formulating a national cancer policy, including cervical cancer screening. An autonomous agency would need to establish sub-units across various districts in Bangladesh. The “National Strategy for Cervical Cancer Prevention and Control Bangladesh” was proposed to mitigate morbidity, disability, and mortality [54]. Effective execution of this strategy necessitates a dedicated institution alongside the national cancer registry. The National Strategy proposes three prevention strategies: (1) primary prevention through HPV vaccination, (2) secondary prevention via screening and treatment of precancerous lesions, and (3) tertiary prevention through early detection and appropriate treatment [54]. While the HPV vaccine is cost-effective for high-income countries [55], its cost-effectiveness for low and middle-income countries remains unexplored. However, before the Bangladeshi government plans, it should investigate the cost-effectiveness and implementation process of including the HPV vaccine in the prevention program by an independent research organization. The current prevention program should emphasize cervical cancer awareness and encourage higher physical activity among women. Initial strategies should include mass media campaigns to raise awareness and promote physical activity, as studies have shown that regular physical activity reduces the risk of developing CC [56, 57]. Developing an effective strategy requires involving public health specialists from various backgrounds and conducting thorough research through independent organizations. The Ministry of Social Welfare can lead the establishment of the national cancer registry, with support from the Ministry of Health and Family Welfare for diagnosis and treatment processes. Distribution of workload will allow the Ministry of Health and Family Welfare to focus on improving the overall health system across Bangladesh. The national strategy also proposed to incorporate the HPV vaccination program with the National Expanded Program on Immunization (EPI), which targets girls aged 9–13; our study indicates that cervical cancer is prevalent among women aged 30–44, with higher screening likelihood among women aged 18–29. Therefore, vaccination efforts should focus on these groups. Additionally, including vaccination programs in the EPI may be costly for a developing country like Bangladesh. Before incorporating the vaccination program in EPI, a large-scale cost-effectiveness study and analysis are required. Our study revealed that women in Barisal had higher screening rates for cervical cancer. In contrast, the Rajshahi division did not report the prevalence of cervical cancer due to participant non-response. This suggests either insufficient training of data collectors or a lack of awareness among women about cervical cancer. The study also revealed whether a woman lived in a rural or urban area, which was a significant predictor, highlighting the need to develop healthcare professionals for both areas. The World Health Organization (WHO) stresses the importance of sustainable cervical cancer detection and management in response to increasing demand [58]. Additionally, specialized training for healthcare professionals, improved infrastructure, and better accessibility to care for economically disadvantaged cervical cancer patients or those residing far from healthcare facilities are needed.

## Conclusion

The proportion of Bangladeshi women who have ever been screened for cervical cancer is low. Future studies should delve into other factors, including socioeconomic status, cultural views, and healthcare access, that influence cervical cancer screening trends for the different divisions in Bangladesh. Establishing an independent national cancer registry to monitor screening rates and outcomes is a pressing need. This will highlight policy success and guide future research and policy decisions.

## Data Availability

Available at https://extranet.who.int/ncdsmicrodata/index.php/catalog/770.
